# Microsatellites for the Marsh Fritillary Butterfly: *De Novo* Transcriptome Sequencing, and a Comparison with Amplified Fragment Length Polymorphism (AFLP) Markers

**DOI:** 10.1371/journal.pone.0054721

**Published:** 2013-01-21

**Authors:** Melanie R. Smee, Yannick Pauchet, Paul Wilkinson, Brian Wee, Michael C. Singer, Richard H. ffrench-Constant, David J. Hodgson, Alexander S. Mikheyev

**Affiliations:** 1 Department of Biology, University of York, York, United Kingdom; 2 Centre for Ecology and Conservation, School of Biosciences, University of Exeter, Penryn, Cornwall, United Kingdom; 3 Max Planck Institute for Chemical Ecology, Jena, Germany; 4 School of Biological Sciences, University of Bristol, Bristol, United Kingdom; 5 National Ecological Observatory Network Incorporated, Washington, D. C., United States of America; 6 Section of Integrative Biology, University of Texas at Austin, Austin, Texas, United States of America; 7 Okinawa Institute for Science and Technology, Onna-son, Kunigami-gun, Okinawa, Japan; Oxford Brookes University, United Kingdom

## Abstract

**Background:**

Until recently the isolation of microsatellite markers from Lepidoptera has proved troublesome, expensive and time-consuming. Following on from a previous study of Edith's checkerspot butterfly, *Euphydryas editha*, we developed novel microsatellite markers for the vulnerable marsh fritillary butterfly, *E. aurinia*. Our goal was to optimize the process in order to reduce both time and cost relative to prevailing techniques. This was accomplished by using a combination of previously developed techniques: *in silico* mining of a *de novo* assembled transcriptome sequence, and genotyping the microsatellites found there using an economic method of fluorescently labelling primers.

**Principal Findings:**

In total, we screened nine polymorphic microsatellite markers, two of which were previously published, and seven that were isolated *de novo*. These markers were able to amplify across geographically isolated populations throughout Continental Europe and the UK. Significant deviations from Hardy-Weinberg equilibrium were evident in some populations, most likely due to the presence of null alleles. However, we used an F_st_ outlier approach to show that these markers are likely selectively neutral. Furthermore, using a set of 128 individuals from 11 populations, we demonstrate consistency in population differentiation estimates with previously developed amplified fragment length polymorphism (AFLP) markers (r = 0.68, p<0.001).

**Significance:**

Rapid development of microsatellite markers for difficult taxa such as Lepidoptera, and concordant results with other putatively neutral molecular markers, demonstrate the potential of *de novo* transcriptional sequencing for future studies of population structure and gene flow that are desperately needed for declining species across fragmented landscapes.

## Introduction

Microsatellite markers are an important tool in population genetics and conservation biology. They typically have high levels of polymorphism and generate reliable data on genetic diversity, population structure and gene flow in highly protected species [1], [2]. At the same time, microsatellites can be amplified from trace amounts of DNA, and permit non-lethal and non-invasive sampling. Although many new genotyping techniques have become available, microsatellites will likely remain relevant for the foreseeable future, given their low input DNA requirements and power for producing reliable genotypes suitable for calculation of familial relationships and for population genetic studies. For many species the usual route to isolating and characterizing microsatellite markers involves a time-consuming process of enriching a genomic DNA library for a variety of di-, tri- or tetra-nucleotide repeat motifs, followed by a process of cloning and sequencing, to finally design species-specific primers [3]–[5]. The design and thorough testing of optimal multiplexes then requires the purchase, for each locus being genotyped, of an expensive primer carrying a fluorescent dye label. Despite this cost, microsatellites remain one of the most popular and useful molecular markers for studies of population structure and conservation decision-making. Capillary DNA sequencers automate microsatellite processing, permitting up to 96 samples to be run simultaneously. Scoring of alleles can likewise be automated, although human verification remains necessary.

Microsatellites often need to be isolated *de novo* when a sequenced genome is unavailable. Although a wide variety of approaches for isolating microsatellites exist [6] they fail frequently with lepidopteran genomes [7]–[10]. This inherent difficulty may be associated with a lack of polymorphism, similarity in flanking regions of different microsatellite loci, presence of null alleles or possible associations with mobile elements [7]–[9], [11], [12]. However, there are still a large number of studies detailing the isolation of microsatellite markers from Lepidoptera [13]–[18]. Inevitably it seems, these studies usually show a low number of loci, low levels of polymorphism, high occurrence of null alleles, or strong deviations from Hardy-Weinberg equilibrium (HWE) [12]–[14], [16], [19]. Often, it is also the case that the developed markers prove not to be transferable between geographically distant populations of the same species. For example, in a recent study on the marsh fritillary butterfly, *Euphydryas aurinia*, three out of an initial 96 loci showed evidence of transferability among geographically distant populations and sub-species [20]. In studies where investigations into geographical patterns of population differentiation are the main aim, such markers cannot be used.

The recent advent of next generation sequencing and the resulting increase in publicly available expressed sequence tag (EST) data has encouraged quick and easy isolation of microsatellite markers [15], [20]–[22]. With the future cost of DNA sequencing decreasing at a rate exceeding Moore's law, the methods used here are becoming much more accessible to a wider research community. However, microsatellites associated with expressed regions of the genome may be under selection, even if they represent untranslated regions (UTRs) of genes, and hence often show strong deviations from HWE [15], [21]. Fortunately, studies that have compared EST-derived microsatellites with other genotyping methods have generally found similar results in studies of population genetics [15], [23], [24].

*E. aurinia* populations have severely declined in both the UK and continental Europe [25]–[27]. Therefore, a current need exists for tools to illuminate population genetic structure throughout this species, in order to aid the setting of conservation priorities. To this end, we report the characterization of seven novel microsatellite loci using next-generation 454-pyrosequencing methods and confirmation of two previously published loci for *E. aurinia*. These markers give concordant results for estimates of population differentiation with amplified fragment length polymorphism (AFLP) markers on the same samples [28]. Their ability to amplify across large geographical regions is also demonstrated. We therefore confirm that these new markers are suitable for use in a conservation genetics study with the aim of informing priorities and targets by determining population structure and gene flow across a fragmented landscape.

## Materials and Methods

RNA was extracted from a pool of 64 *E. aurinia* larvae collected from 16 sites in south-west UK (under Natural England licence number 20081071), using TRIzol (Invitrogen). Genomic DNA contamination was removed by DNase treatment (TURBO DNase, Ambion) for 30 min at 37°C. RNA was further purified by using the RNeasy MinElute Clean up Kit (Qiagen) following the manufacturer's protocol. Full-length, enriched, cDNAs were generated from 2 µg of total RNA using the SMART PCR cDNA synthesis kit (BD Clontech) following the manufacturer's protocol. Reverse transcription was performed with the PrimeScript reverse transcriptase (Takara) for 60 min at 42°C and 90 min at 50°C. In order to prevent over-representation of the most common transcripts, the resulting double-stranded cDNAs were normalized using the Kamchatka crab duplex-specific nuclease method (Trimmer cDNA normalization kit, Evrogen) [29]. The resulting normalized cDNA library was sent to the Advanced Genomics facility at the University of Liverpool (http://www.liv.ac.uk/agf) for sequencing on the Roche 454 GS-FLX pyrosequencing platform. The assembly of the obtained reads was achieved using MIRA v2.9.26×3 (http://sourceforge.net/apps/mediawiki/mira-assembler/index.php?title=Main_Page). The mira assembler was run using a perl wrapper script (EST2assembly [30]) which parameterizes and selects the optimal assembly, based on coverage, redundancy and the proportion of coverage to a reference transcriptome. The mira command line for the optimal assembly was: mira-job = denovo, est, accurate,454–fasta-OUT: rrol = 1:rld = 1:orc = 1:org = 0:ora = 0:ors = 0:otf = 0:otc = 0–GE:not = 1-CO:asir = 1-LR:mxti = 1-AS:sd = 0:uess = 0:urd = 0:ard = 1-SKIM:mmhr = 2:mnr = yes 454_SETTINGS-DP:ure = 0-CO:mrpg = 10-CL:pvlc = 0:cpat = 0:mbc = 1:mbcgs = 30:mbcmfg = 30:mbcmeg = 30:qc = 0-ED:ace = 1-AL:egp = no-ALIGN:bip = 20:bmax = 120:mo = 10:mrs = 80.

The assembled data were queried for ≥5 perfect repeats of di-, tri- and tetra-nucleotide repeats using Msatfinder version 2.0.9 [31]. Primers were then designed using Primer3 for 74 contigs containing microsatellites [32]. Forward primers were designed with an addition of a M13 tail at the 5′ end (M13: 5′-TGTAAAACGACGGCCAGT-3′) following the methods of Schuelke [33]. This allows the use of fewer expensive fluorescently labelled primers, since only one ‘universal’ fluorescently labelled M13 primer is required per multiplex. PCR conditions are such that a small quantity of unlabelled sequence-specific forward primer with a M13 tail is incorporated first, in partnership with a sequence-specific reverse primer. Once the forward primer is depleted, the annealing temperature is lowered so that the ‘universal’ fluorescently labelled M13 forward primer anneals instead (its annealing temperature is a few degrees lower). The fluorescent dye is thus incorporated into the PCR product [33]. A differently labelled M13 primer can be used for each multiplex and then all multiplexes can be combined when sequencing. We also synthesized previously published primers of *E. aurinia* microsatellite markers [16], [34] with the addition of a M13 tail and tested these on our samples.

*E. aurinia* (Lepidoptera; Nymphalidae) is a univoltine species, with gregarious larvae [35]. A single 3rd/4th instar larva was collected from each of 281 communal webs found in September 2008 and 2009 (Natural England licence numbers 20082745 and 20091722; Countryside Council for Wales licence OTH:SPCA:09:2008; and Scottish Natural Heritage licence 9154) from a total of 16 sites spanning the butterfly's mainland UK range (Figure S1). Each larva collected from south-west UK, Scotland and Wales was starved for 24 hours to remove plant material from the digestive tract and then dissected to remove the midgut and potentially any parasitoids, before then being snap frozen. Following the methods of Martinez-Torres *et* *al*. [36] with slight modification (38 μl of 3 M potassium acetate, pH 5.2, was used and the samples then incubated at −80°C for 15 mins), genomic DNA was extracted, diluted to a working concentration (∼15 ng/µl), and stored at −20°C until required. DNA from samples collected from 12 sites in France and Catalonia (Figure S1) was extracted from the heads of larvae using DNeasy Tissue kits (Qiagen) [28]. All 74 designed primer pairs were initially tested for amplification on four samples from geographically widespread populations in the UK (site numbers 1, 6, 8 and 11: see Table S1 and Figure S1 for details and geographical distribution of all populations used in the study). Each 10 µl PCR mix contained approximately 1.5 ng genomic DNA, 1 µl 10x EX Buffer (Takara), 2 µM dNTPs, 0.25 U EX Taq HS (Takara), 1 µl 10 mg/ml BSA, 10 pmol fluorescently labelled M13 forward primer, 10 pmol reverse primer and 2.5 pmol of the sequence specific forward primer. Using a PTC-200 Thermo Cycler (MJ Research) the PCR conditions were as follows: 95°C for 5 mins; followed by 25 cycles of 95°C for 5 s, 60°C for 30 s, 68°C for 1 min; followed by 8 cycles of 95°C for 5 s, 53°C for 30 s, 68°C for 1 min; and ended with a final extension at 72°C for 30 mins. A maximum of 2 µl of PCR product was then added to 10 µl HiDi formamide (Applied Biosystems) and 0.1 µl GeneScan 500 ROX standard (Applied Biosystems) and run on an ABI 3130xl Genetic Analyzer (Applied Biosystems). Allele presence and sizes were assigned using GeneMarker 1.7 (SoftGenetics, LLC).

Potentially polymorphic loci were then tested on a further four samples from different populations in the UK (site numbers 9, 12, 13 and 14: see Table S1 and Figure S1). For loci that amplified across all eight samples and appeared polymorphic, we designed multiplexes using PCR products with non-overlapping size ranges. Multiplexes using differently labelled M13 ‘universal’ forward primers were then mixed together and analyzed on an ABI 3100 capillary sequencer (on average 0.5 µl of each multiplex was added to the ROX and HiDi formamide mix, but this was adapted according to the intensity of the signal). All microsatellite sequences were submitted to GenBank under accession numbers JN116271 and JN116273 to JN116283.

GENEPOP v4.1 was used to assess deviations from HWE [37]. Estimates of null allele frequencies were achieved following the Expectation Maximization (EM) algorithm of Dempster *et* *al* [38] in the online programme FreeNA [39]. The programme LOSITAN was then used to identify putative loci under selection across our samples, using a method of comparing *F*_ST_ values against expected heterozygosity (*H*_E_) to detect outliers [40], [41]. As a subset of samples (N = 128) had been previously genotyped using AFLP markers [28], the concordance between this previous analysis and the current study was assessed by comparing the Φ_st_ and F_st_ matrixes generated by the two studies, respectively, using a Mantel test with 10,000 bootstrap replicates. Both Φ_st_ and F_st_ matrixes were computed using Arlequin v3.5 [42].

## Results

**Figure 1:**
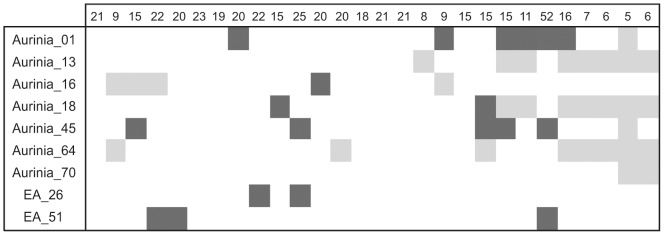
Hardy-Weinberg equilibrium (HWE) statistics. ‘Exact test’ statistics for each locus, and for each of the 28 populations included in the study (each column represents a population with sample size in the top row, with the populations occurring in the same order, left to right, as numbered in Figure S1 and Table S1), after a sequential Bonferroni correction for multiple tests [43]. Dark shading indicates significant deviation from HWE (chi-squared test, p<0.05); light grey shading indicates monomorphic loci, or only a single copy of a second allele if present. For loci with less than five alleles the complete enumeration method was used [51] but for all others a Markov chain algorithm was implemented [52] in GENEPOP v.4.1 [37].

The 454 run generated 186,835 reads, and a total of 40,865,774 bases, after quality filtering. These were assembled into 22,032 contigs whereby 50% were equal to or larger than a size of 932 bases (N50 size) resulting in an average 3.15-fold coverage. Primers were designed for 74 microsatellite loci out of the 97 found in the 454 transcriptome, as some were too close to the edge of the contig for primer placement. Across an initial sample of four individuals, 36 of the novel loci did not amplify at all, 24 appeared monomorphic and 14 were potentially polymorphic. Out of the eleven previously published loci, only two appeared polymorphic in our samples. Testing across another four samples resulted in discarding of two of the novel primers due to a lack of amplification across all samples, and possible null alleles (whereby a mutation in the primer annealing site prevents amplification). The remaining loci (see Table 1) were developed into multiplex sets to enable all samples to be genotyped in just two sequencing mixes. The locus Aurinia_14 was discarded during multiplex design as it did not successfully amplify with other primer combinations, or even reliably amplify alone. Loci Aurinia_23B, Aurinia_31B, Aurinia_62 and Aurinia_35 either did not amplify well or gave large amounts of missing data, and thus were excluded from further analyses leaving nine loci for large-scale genotyping. In many of the sampled populations there was significant deviation from HWE even after sequential Bonferroni correction for multiple tests [43] (Figure 1).

**Table 1:** Characteristics of 14 microsatellite loci in *Euphydryas aurinia* (Lepidoptera: Nymphalidae).

PCR #	Locus	Primer sequences (5′ to 3′)	M13 Label	Repeat motif	Range (bp)	N_A_	H_O_	H_E_	% missing	Average % null alleles	GenBank Acc. no.
1	Aurinia_01	F:CGAGCGTATTTGTCAAAGAAAG	HEX	(CAT)_6_	252–270	6	0.396	0.503	8.1%	10.4	JN116271
		R:AGCGAATTAGGGTTGTCACATT									
2	Aurinia_13	F:AACGTTAACACTAGGGGTCTCA	TET	(ATT)_6_	228–237	4	0.332	0.294	0.85%	0.9	JN116273
		R:TATGATATAGTGTACGCGGTTTTT									
2	Aurinia_16	F:CCCGCTATGATCCATGTTTTA	TET	(TTA)_6_	173–204	6	0.343	0.367	0.2%	4.6	JN116275
		R:AAATTCATTTGTACTTTCGGTACAT									
4	Aurinia_18	F:AAAAGCGCTGAAAGAAGAAAAA	TET	(TAT)_5_	189–193	4	0.327	0.384	1.1%	4.0	JN116276
		R:CAGTCTCAAAGATTTCGCATAAAA									
1	Aurinia_45	F:GGGTGAAATTGCGAATGAGT	HEX	(GTT)_6_	193–213	8	0.452	0.566	3.4%	8.9	JN116280
		R:TCCCCGCTACAGATGAAATC									
3	Aurinia_64	F:CAACCTGTAGCCGGAAAAGA	TAM	(TAC)_8_	201–210	4	0.188	0.203	4.0%	3.5	JN116282
		R:GCTTTTCTGTTGCCATCGTT									
1	Aurinia_70	F:CAACTTCAGTATGATCTCATTGCTTT	HEX	(GA)_10_	130–144	8	0.431	0.370	1.5%	1.1	JN116283
		R:TCACAATTTGCAGTGGCTCTAT									
1	EA26*	F:CCGAGATACTCACCTACAAG	HEX	(TG)_5 _TA(TG)_11_	163–180	13	0.705	0.730	5.7%	4.2	AY491806
		R:CAGTGTATTTCGGAACACAG		(AGCG)_4 _(TG)_3_							
5	EA51*	F:TGACGACAGATGGGTGTTC	HEX	(TGTA)_9 _(TGAT)_2_	129–142	4	0.483	0.622	5.1%	11.9	AY491828
		R:TGTAAGCGACTCAGTCTCATTTC									
-	Aurinia_14	F:TTTGTATGGGGAGAATTTATTGTTT	-	(AT)_8_	226–232	4	-	-	-	-	JN116274
		R:TTTCTTTTAATCACAGATAACCTTTTT									
3	Aurinia_23	F:TACAAGCGCTTACCGAAGAAAC	TAM	(GAT)_7_	147–162	5	-	-	-	-	JN116277
		R:TCTGTCTGTTCATCGCTCTCA									
2	Aurinia_31	F:CAATTTAGGCGGCAAATTAAGA	TET	(AAC)_5_	252–258	2	-	-	-	-	JN116278
		R:CCCGAGCTAAGCGACTACACTA									
2	Aurinia_35	F:CAAGAACATGAATTTAGGTAAGCA	TET	(TAT)_5_	122–130	4	-	-	-	-	JN116279
		R:CATAAGATTATGCCGGTATATAAAGTT									
1	Aurinia_62	F:TGTAGGCGACGTTCTTGCTA	HEX	(AAT)_5_	240–243	2	-	-	-	-	JN116281
		R:AATGCATTTTCGCATTCTAGG									

Summary statistics based on a survey of 468 individuals from 28 populations. N_A_, number of observed alleles; H_O_ observed and H_E_ expected heterozygosity per locus. All Forward primers had M13 tail (TGTAAAACGACGGCCAGT) added to 5′ end, as described in text. Loci below the line were lost due to a lack of amplification across all samples, or because of null alleles, but may prove of use in other populations or closely-related species. PCR reactions 1, 2 and 3 were pooled and analyzed together in the same sequencer run, as were reactions 4 and 5– see text for description. *Primers were originally published by Petenian *et* *al*. [16].

**Figure 2:**
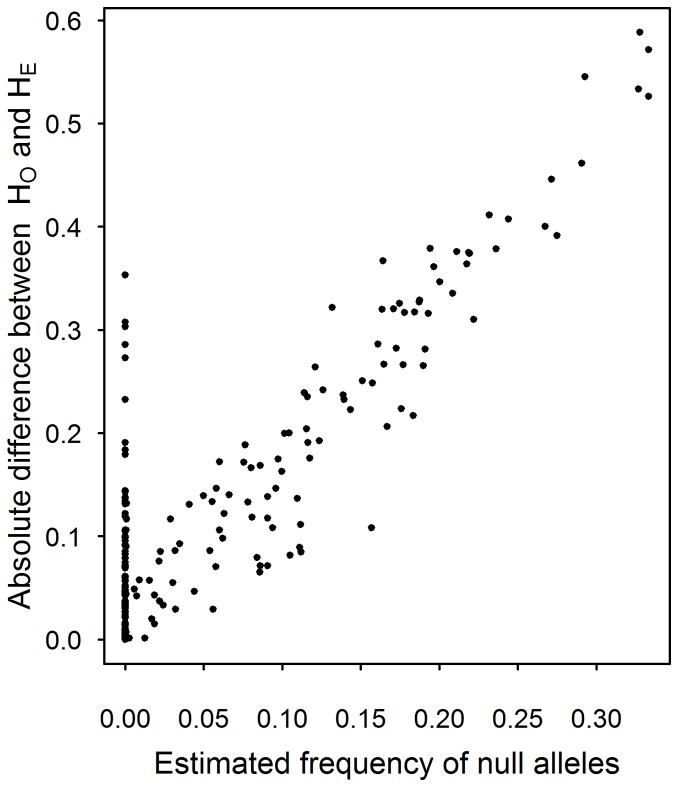
Deviation from HWE and estimated frequency of null alleles. The absolute difference between expected and observed heterozygosity in a population, as a measure of deviation from HWE, plotted against the estimated frequency of null alleles per marker per population for all nine loci to be used in future population genetic studies. Each data point represents one locus in a specific population.

These deviations from HWE are likely due to the presence of null alleles in many cases. Analyses using FreeNA [39] gave estimated null allele frequencies ranging from 0 to 0.33 per marker per population, which were highly correlated with the absolute difference between expected and observed heterozygosities – a measure of deviation from HWE (Figure 2).

**Figure 3:**
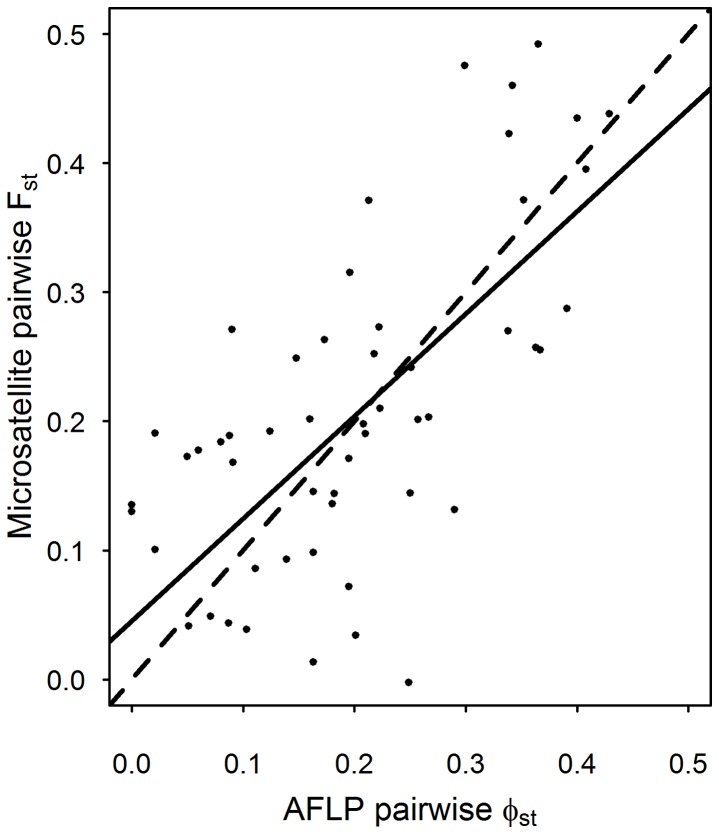
A comparison of microsatellite and AFLP markers on the same samples. A comparison of pairwise population differentiation (uncorrected F_st_ and Φ_st_) estimated using the seven novel microsatellites developed in the current study and AFLP markers developed in a previous study by Wee [28] on the same set of 11 European populations (solid line). A dashed line is also included to show where a perfect correspondence between the two would lie.

The extent to which deviations from HWE may affect typical analyses using EST-derived markers is unclear. We were able to validate our markers using AFLP-derived distances computed for the same samples in an earlier study, despite the presence of null alleles [28]. Hence, we show very similar results for estimates of pairwise population differentiation using only the seven novel microsatellite loci developed in this study and AFLP markers developed previously [28] on a subset of the populations analysed here (Mantel test: r = 0.68, n = 11, p<0.001, Figure 3). Adding the two previously developed microsatellite loci, EA26 and EA51 (Table 1), further strengthens this relationship (Mantel test: r = 0.72, p<0.0005). The occurrence of null alleles can be corrected for when calculating *F*_st_ and genetic distances in studies of population structure [39]. However, this correction does not explain any significant geographic variation in population structure, relative to the uncorrected matrix (partial Mantel test of geographic distance and the corrected Fst matrix, using the un-corrected matrix as the covariate; p = 0.23), meaning our results are robust and do not change significantly after correction.

**Figure 4:**
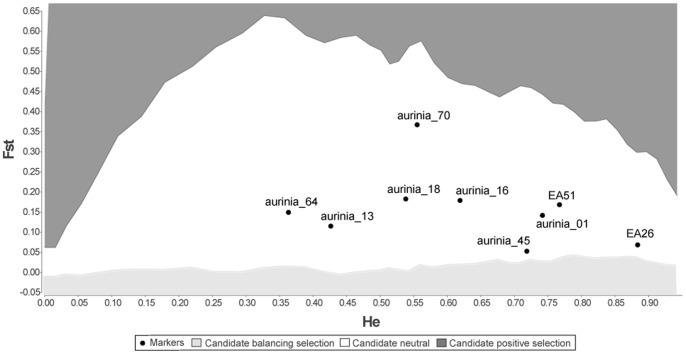
LOSITAN results for all nine loci across all samples. This workbench uses an F_st_ outlier method to detect potential loci under selection and therefore unsuitable for use in studies reliant on neutral markers, such as population genetics. Under a stepwise mutation model (SMM), all loci were found to be candidate neutral markers – located in the white region of the figure. The dark grey region above indicates where candidate positive selection markers would be found, and the lighter region below indicates where candidate balancing selection markers would be found. Under an infinite alleles model (IAM) of mutation, the result is the same.

We were also able to demonstrate that all loci fell into the candidate neutral category after using the programme LOSITAN [40], [41] to identify putative loci under selection across all our samples (Figure 4). Four pairs of loci showed evidence of linkage disequilibrium (LD) across all populations (EA26-Aurinia_45, EA26-Aurinia_01, EA51-EA26, EA51-Aurinia_45) however, following a sequential Bonferroni correction only one remained marginally significant (EA51-Aurinia_45).

## Discussion

This study has successfully isolated and characterized microsatellites for a threatened lepidopteran species using a rapid method combining data mining for perfect repeats with inexpensive primer labelling. Schuelke's [33] M13 primer protocol has particular advantages for small research groups performing low-throughput genetic analyses, by reducing costs of fluorescently labelled primers, particularly useful for initial primer screening, where primers may be used possibly only once.

Effective and reliable DNA markers are desirable for taxa with important roles in agriculture, ecology and horticulture, such as the Lepidoptera. The markers developed in this study originated from a 454-pyrosequencing run using first-generation chemistry, and hence likely do not represent the entire breadth of markers to be found. The low coverage of the transcriptome and the potential for assembly errors, especially where there are homopolymer runs, may explain why so many of the initially designed primers failed to amplify. There were also time constraints, which highlights the efficiency of our approach, taking a full time researcher only two months from receiving primers to analysis.

This study also confirmed the successful use of two established microsatellite loci for the study species, although they both showed considerable deviations from HWE across the sampled populations [16]. Such deviations in HWE, aside from being the result of null alleles, can be expected in *E. aurinia* and other Lepidoptera with similar life histories, for several biologically plausible reasons. Butterflies in the subfamily Melitaeinae, to which the genus *Euphydryas* belongs, typically suffer frequent population extinctions and their persistence in a landscape can depend entirely on metapopulation dynamics [44]. Although population structure is often discrete [45] and most individuals travel very short distances in their lives, occasional longer distance dispersal events occur [46], [47]. In *E. aurinia*, mean clutch size is several hundred eggs [48], such that each female is unlikely to lay more than two clutches in her lifetime. Effective population size is reduced by the correlated survival of siblings that results from this large clutch size and by extreme population fluctuations [49]. Also affecting HWE expectations are the high levels of inbreeding found in small subpopulations within metapopulations [50], which is an unavoidable result of habitat fragmentation and stochastic patch dynamics. Our final analysis using LOSITAN [40], [41] confirms that all loci are putatively neutral, and so deviations are likely to be due to actual population structure and dynamics, but also, significantly, due to the presence of null alleles across the markers themselves. Studies using these markers to compute Hardy-Weinberg equilibrium should make corrections for the presence of null alleles, although F_st_-based measures of population differentiation appear relatively robust (see also Mikheyev *et* *al* [15]).

The markers developed here from a 454 transcriptome fulfil a large number of desired criteria for developing ‘robust’ microsatellite markers for *E. aurinia* [20]. This includes perfect repeat motifs (neither compound microsatellites nor with any interruptions), being PCR ‘multiplexable’, and being transferable between geographically distant populations [20]. The one caveat, or unfulfilled criterion, is that some loci in some populations may have substantial numbers of null alleles. Sinama *et* *al* [20] recently developed microsatellite markers for *E. aurinia* using a combination of a biotin-enrichment protocol and next generation pyrosequencing on genomic DNA, resulting in three markers without the presence of null alleles. These three markers also showed transferability between populations geographically further apart than those used in this current study, and between sub-species of *E. aurinia* [20]. Although null alleles can overestimate the genetic distance in significantly differentiated populations [39], the extent to which they affect the measurement of population differentiation in *E. aurinia* is unknown *a priori*. Furthermore, there is no guarantee that some population not sampled during marker development will not have null alleles. Aware of this problem in our markers, we validated their use for estimating genetic distances between populations with data from AFLP markers, with and without correction for the presence of null alleles [39]. Sinama *et* *al* [20] did not have AFLP markers as a comparison. However that study rejected four microsatellite markers because of deviations from HWE in only one out of the three populations tested. Such markers might actually prove usable if they could be validated. Approaches such as those used in both this study and by Sinama *et* *al* [20], may both be useful for efficient and cost effective development of robust markers for any endangered or non-model lepidopteran species, and should be the methods of choice for future studies. Marker validation through an alternative technique remains a plus.

## Supporting Information

Figure S1 **Map showing location of all 28 populations sampled in the present study.** Populations 18 to 28 are those also included in a previous study using AFLP markers [28].
(TIF)

Table S1 **Sites across the UK and Catalonia region of Europe from which** ***E. aurinia*** **was sampled.**
(DOCX)
