# Supplementary figures and images for: Microsatellites for the Marsh Fritillary Butterfly: De Novo Transcriptome Sequencing, and a Comparison with Amplified Fragment Length Polymorphism (AFLP) Markers
Source: PLoS One. 2013 Jan 21;8(1):e54721. doi: 10.1371/journal.pone.0054721 (PMC3549983; doi:10.1371/journal.pone.0054721)

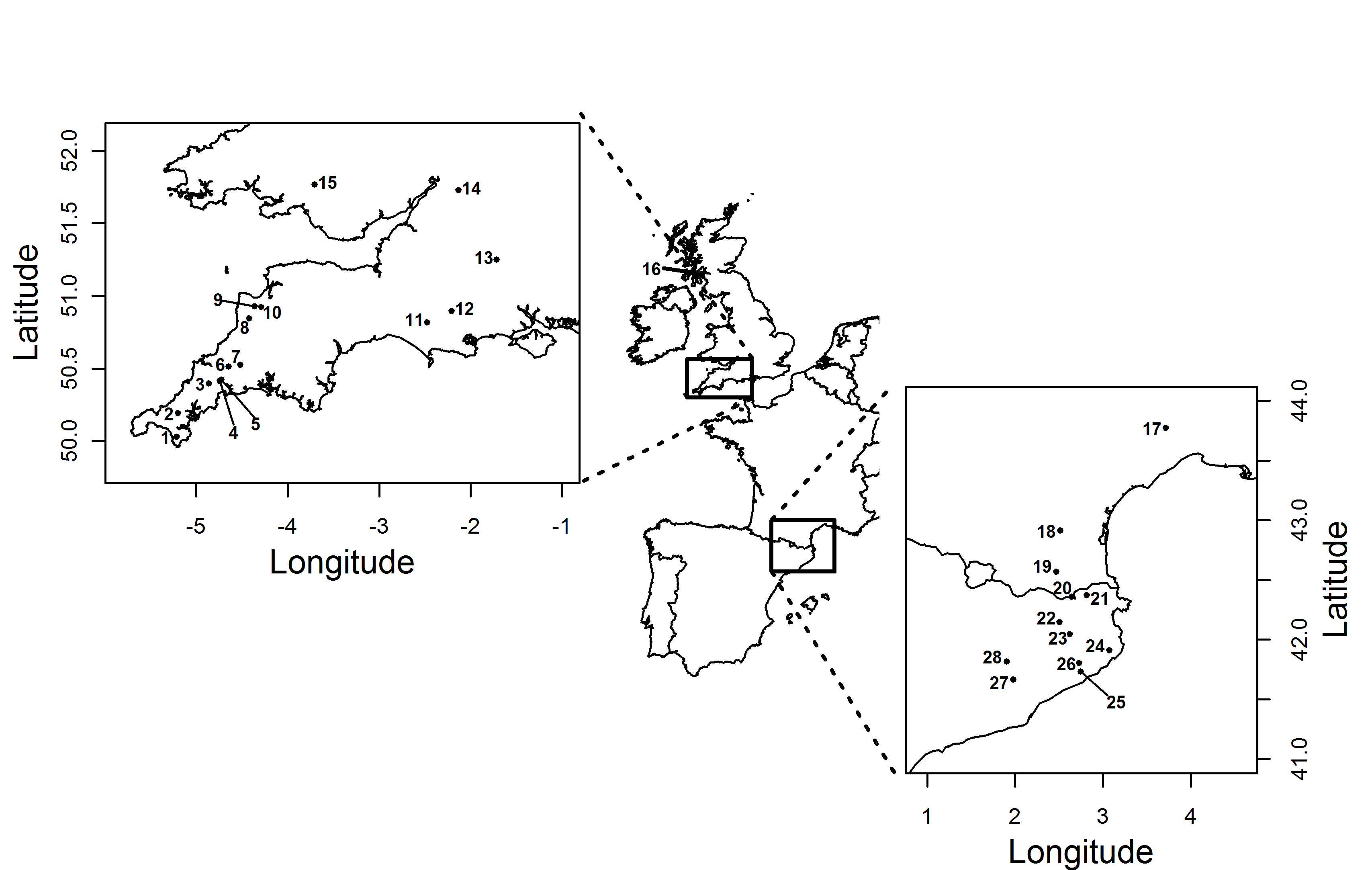

Supplement: Figure S1 — Map showing location of all 28 populations sampled in the present study. Populations 18 to 28 are those also included in a previous study using AFLP markers [28]. (TIF) [file pone.0054721.s001.tif]
