# Supplementary material for: Microsatellites for the Marsh Fritillary Butterfly: De Novo Transcriptome Sequencing, and a Comparison with Amplified Fragment Length Polymorphism (AFLP) Markers
Source: PLoS One. 2013 Jan 21;8(1):e54721. doi: 10.1371/journal.pone.0054721 (PMC3549983; doi:10.1371/journal.pone.0054721)
Supplement: Table S1 — Sites across the UK and Catalonia region of Europe from which E. aurinia was sampled. (DOCX) [file pone.0054721.s002.docx]

| Site # | Area/County | Population Name | Lat. | Long. | | Sample Size |
| --- | --- | --- | --- | --- | --- | --- |
| 1 | Cornwall | Lizard | 50.0285 | | -5.2113 | 21 |
| 2 | Cornwall | Stithians | 50.1917 | | -5.1985 | 9 |
| 3 | Cornwall | Goss Moor | 50.3957 | | -4.8590 | 15 |
| 4 | Cornwall | Breney Common | 50.4139 | | -4.7404 | 22 |
| 5 | Cornwall | Redmoor | 50.4215 | | -4.7225 | 20 |
| 6 | Cornwall | Colvannick | 50.5123 | | -4.6473 | 23 |
| 7 | Cornwall | Carkeet | 50.5251 | | -4.5168 | 19 |
| 8 | Devon | Dunsdon | 50.8446 | | -4.4204 | 20 |
| 9 | Devon | Volehouse | 50.9277 | | -4.3592 | 22 |
| 10 | Devon | Stowford | 50.9219 | | -4.2906 | 15 |
| 11 | Dorset | Giants Hill | 50.8175 | | -2.4755 | 25 |
| 12 | Dorset | Hod Hill | 50.8956 | | -2.2075 | 20 |
| 13 | Wiltshire | Salisbury | 51.2479 | | -1.7134 | 20 |
| 14 | Gloucestershire | Strawberry Bank | 51.7274 | | -2.1332 | 18 |
| 15 | Wales | Wales – varied | 51.7655 | | -3.7041 | 21 |
| 16 | Scotland | Scotland - varied | 55.9790 | | -5.6674 | 21 |
| 17 | S. France | Pic St Loup | 43.7715 | | 3.7156 | 8 |
| 18 | S. France | Col de la Redoulade | 42.9122 | | 2.5145 | 9 |
| 19 | S. France | Col del Forn | 42.5681 | | 2.4686 | 15 |
| 20 | S. France | Coustouges | 42.3584 | | 2.6473 | 15 |
| 21 | Catalonia | Darnius | 42.3704 | | 2.8178 | 15 |
| 22 | Catalonia | Can Jorda | 42.1443 | | 2.5038 | 11 |
| 23 | Catalonia | LaBaraca | 42.0457 | | 2.6254 | 52 |
| 24 | Catalonia | Mas Calc | 41.9112 | | 3.0723 | 16 |
| 25 | Catalonia | Tordera | 41.7308 | | 2.7475 | 7 |
| 26 | Catalonia | Sils | 41.8004 | | 2.7310 | 6 |
| 27 | Catalonia | Col Estelales | 41.6635 | | 1.9791 | 5 |
| 28 | Catalonia | El Guix | 41.8159 | | 1.9044 | 6 |
|  |  |  |  | |  |  |
